# Acute upper extremity ischemia and symptomatic popliteal artery aneurysm secondary to coronavirus disease 2019

**DOI:** 10.1016/j.jvscit.2021.02.009

**Published:** 2021-03-02

**Authors:** Karthikeshwar Kasirajan

**Affiliations:** Division of Vascular and Endovascular Surgery, Department of Surgery, University of Stanford, Pleasanton, Calif

**Keywords:** ACE2, Aneurysm, Angiotensin-converting enzyme 2, Arterial thrombosis, COVID-19, SARS-CoV-2

## Abstract

We report the cases of two patients with coronavirus disease 2019 (COVID-19). One patient had presented with acute right upper extremity ischemia (axillary artery thrombosis) and one patient with a symptomatic popliteal artery aneurysm (7.5 × 7.2 cm). Both patients had tested positive for COVID-19 but had no systemic symptoms. The patients were successfully treated using percutaneous techniques and subsequently discharged with oral anticoagulation therapy. A review of the pathogenesis of SARS-CoV-2 (severe acute respiratory syndrome coronavirus 2)–related arterial thrombosis and aneurysmal disease was performed and discussed.

The World Health Organization declared coronavirus disease 2019 (COVID-19), a viral disease caused by severe acute respiratory syndrome coronavirus 2 (SARS-CoV-2), as a pandemic on March 11, 2020.^1^ Although globally the incidence has been >22 million at the time of our writing, the need for vascular surgical interventions specific to COVID-19 has not explored. In the present report, the current understanding of the pathogenesis of thrombosis in patients with COVID-19 has been reviewed. Additionally, it is now recognized that the angiotensin-converting enzyme 2 (ACE2)/angiotensin 1-7/Mas axis is downregulated by SARS-CoV-2.^2^ The downregulation of ACE2 and its role in the pathogenesis of aneurysms was also discussed. Both patients provided written informed consent to report the details and imaging studies of their case.

## Case report

### Patient 1

**Fig 1:**
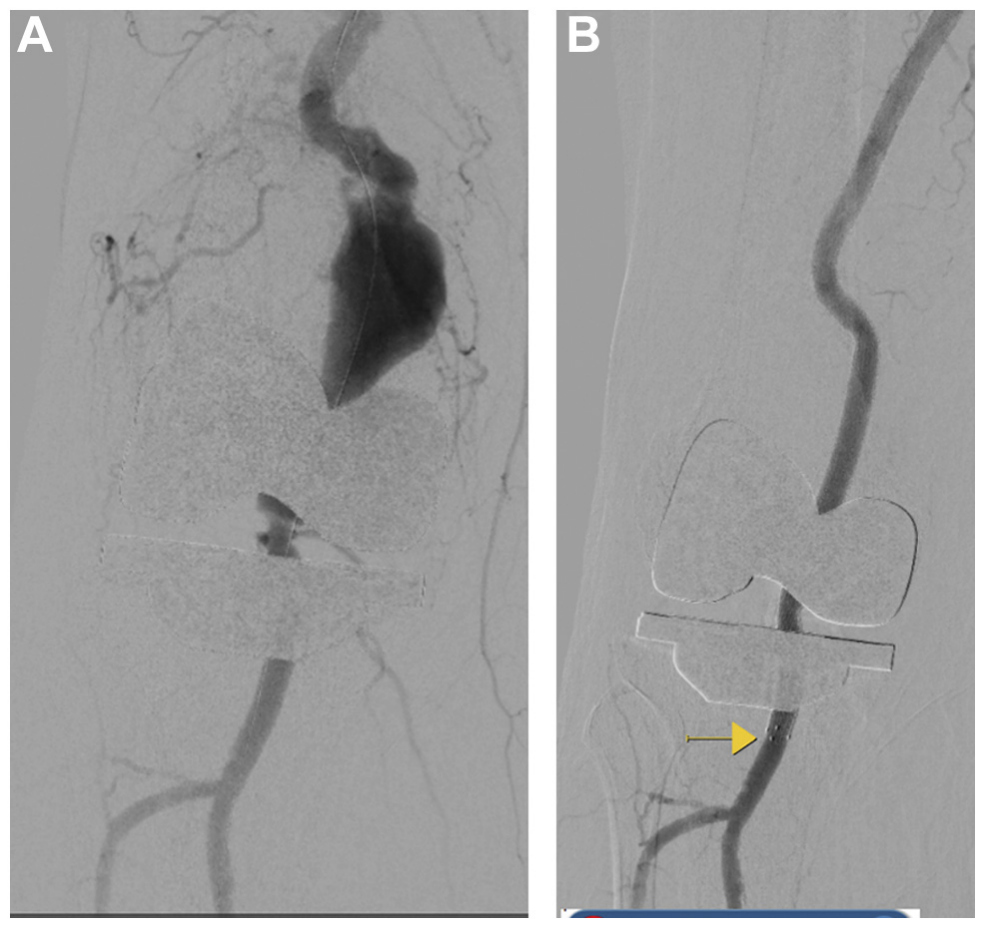
**A,** Diagnostic angiogram before aneurysm exclusion. **B,** Exclusion of the aneurysm with a covered stent. *Arrow* indicates distal extent of the stent.

A 77-year-old man with a significant medical history of hyperlipidemia and hypertension had presented with a 3-day history of new-onset pain, swelling behind his right knee, and distal edema. The patient had undergone right knee replacement 8 years before this presentation. He had not undergone any imaging studies since his knee replacement. Hence, the aneurysm size before COVID-19 was unavailable. Computed tomography angiography (CTA) of the right leg demonstrated a 7.5 × 7.2-cm popliteal artery aneurysm with no evidence of rupture, leak, or distal embolization. Clinically, the patient had a visibly pulsatile mass behind his right knee, with worsening pedal edema and palpable pedal pulses. The inability to bend his knee to >90° owing to the increasing pressure causing pain had been noted just 3 days before presentation. The pain was significantly relieved with full extension. He had no evidence of deep vein thrombosis found by the CTA or foot drop clinically. A nasopharyngeal swab for SARS-Cov-2 RNA test was positive. The patient had no respiratory symptoms or fever. The laboratory test results were all normal, except for elevated C-reactive protein (CRP) and D-dimer levels and a slightly diminished platelet count (Table). Treatment was undertaken using conscious sedation. Contralateral femoral access was obtained, and the aneurysm was excluded using a 8-mm × 250-mm Viabahn stent (W.L. Gore, Flagstaff, Ariz) placed through a 7F sheath (Fig 1). The next day, the patient continued to have palpable pedal pulses with no pulsation over the popliteal aneurysm, and he was discharged home with a prescription for apixaban 5 mg twice daily. At the 1-month follow-up examination, his knee pain had resolved, and duplex imaging studies had demonstrated aneurysm thrombosis with graft patency. The size of the aneurysm was stable compared with previous computed tomography images. At 6 months after treatment, the patient's symptoms had resolved, and he was able to flex his knee to the status before his initial presentation. The patient also noted that he has fewer visible varicose veins compared with previously.

**Table:** Blood parameters for both patients

Variable	D-Dimer, μg/mL	CRP, mg/L	Platelet count, K/μL	Blood group
Aneurysm	6.23	25.2	145	O+
Limb ischemia	8.28	200.1	449	A+
Reference range	0-0.49	<0.5	150-400	NA

*CRP,* C-reactive protein; *NA,* not applicable.

### Patient 2

**Fig 2:**
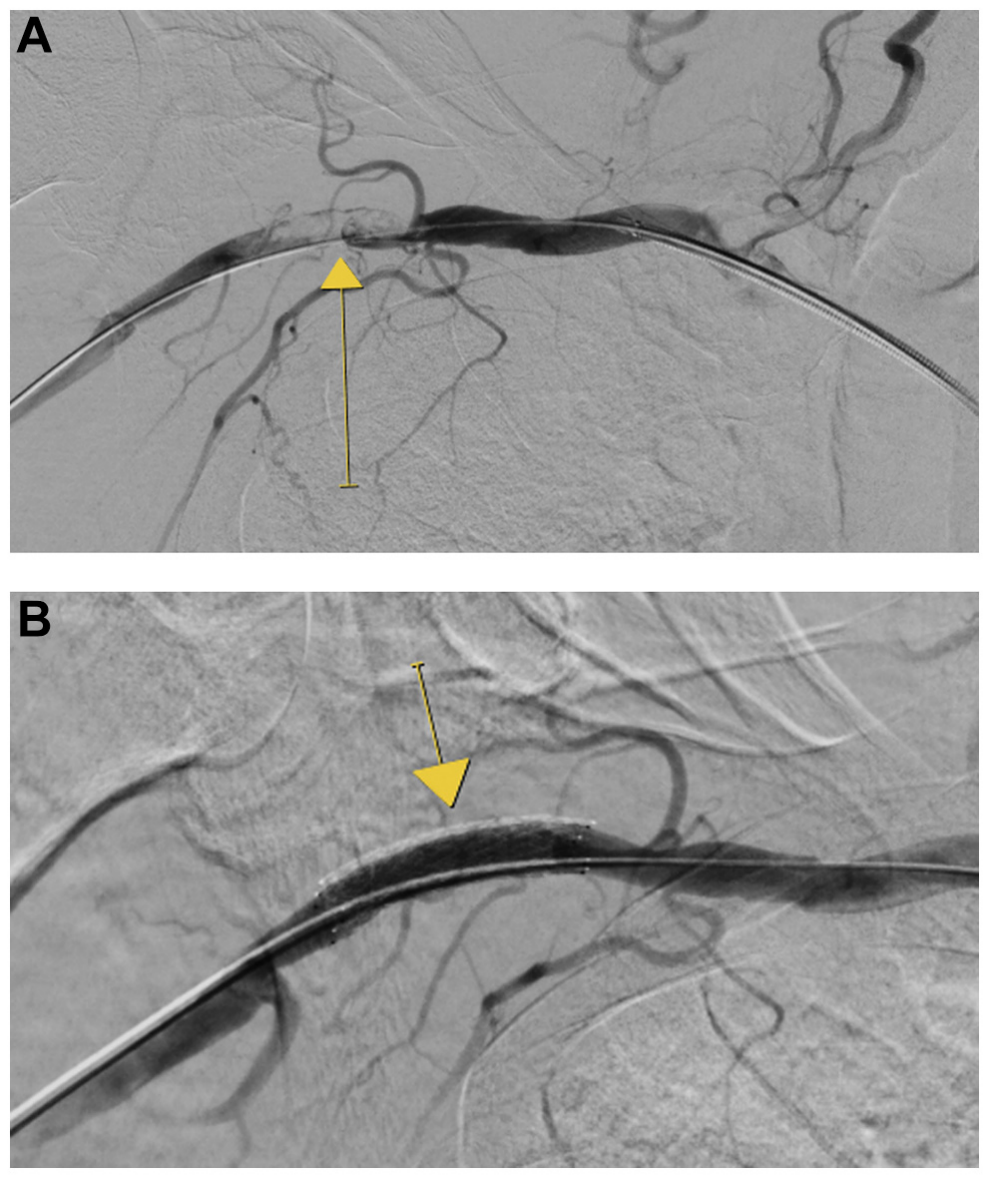
**A,** Angiogram demonstrating in situ axillary artery thrombosis. *Arrow* points to site of thrombosis. **B,** Completion angiogram showing self-expanding stent in place. *Arrow* points to location of treated segment.

A 71-year-old man with a medical history of hypertension and hypothyroidism had presented with a 2-hour history of severe right hand pain. The patient underwent CTA, which demonstrated an axillary artery occlusion with ground-glass opacification in the lungs. Clinically, the patient had no radial or brachial pulses in his right arm, and pallor, hypoesthesia, and coolness of the right arm were present. The patient had had no fever or respiratory symptoms but had had an oxygen saturation of 88% on room air. A nasopharyngeal swab for SARS-CoV-2 RNA test was positive. The patient underwent an emergency right upper extremity angiography, which demonstrated an acute axillary artery thrombosis (Fig 2, *A*). The lesion was crossed with a spider filter (Medtronic Inc, Minneapolis, Minn) and treated with AngioJet (Boston Scientific, Marlborough, Mass) mechanical thrombectomy. Repeat angiography demonstrated minimal residual thrombus, which was managed with a self-expanding stent (Fig 2, *B*). No evidence of vascular compression at the thoracic outlet was noted during provocative maneuvers. The patient developed a palpable radial pulse with resolution of pain. The patient was given oral steroids and apixaban. He was discharged on the fourth postoperative day once he no longer required supplemental oxygen. At his 1-month follow-up examination, he had had continued arterial patency and no new thrombotic events.

## Discussion

Popliteal artery aneurysms will typically be discovered incidentally or will present most often with distal embolization. Giant popliteal artery aneurysms or ruptured popliteal artery aneurysms are rare.^3^ In the largest reported series, ruptured popliteal artery aneurysms were an average of 6.3 cm in diameter. In our report, despite the large size (7.5 cm), the aneurysm had no evidence of rupture. The new-onset pain most likely represented an impending rupture. The unusual event was our patient's inability to bend his knee for the previous 3 days. The patient was an active man, used to working in the garden and was accustomed to working with his knees flexed to >90 degrees on most days. This points to a rapid increase in aneurysm size during the last few days before his presentation.

**Fig 3:**
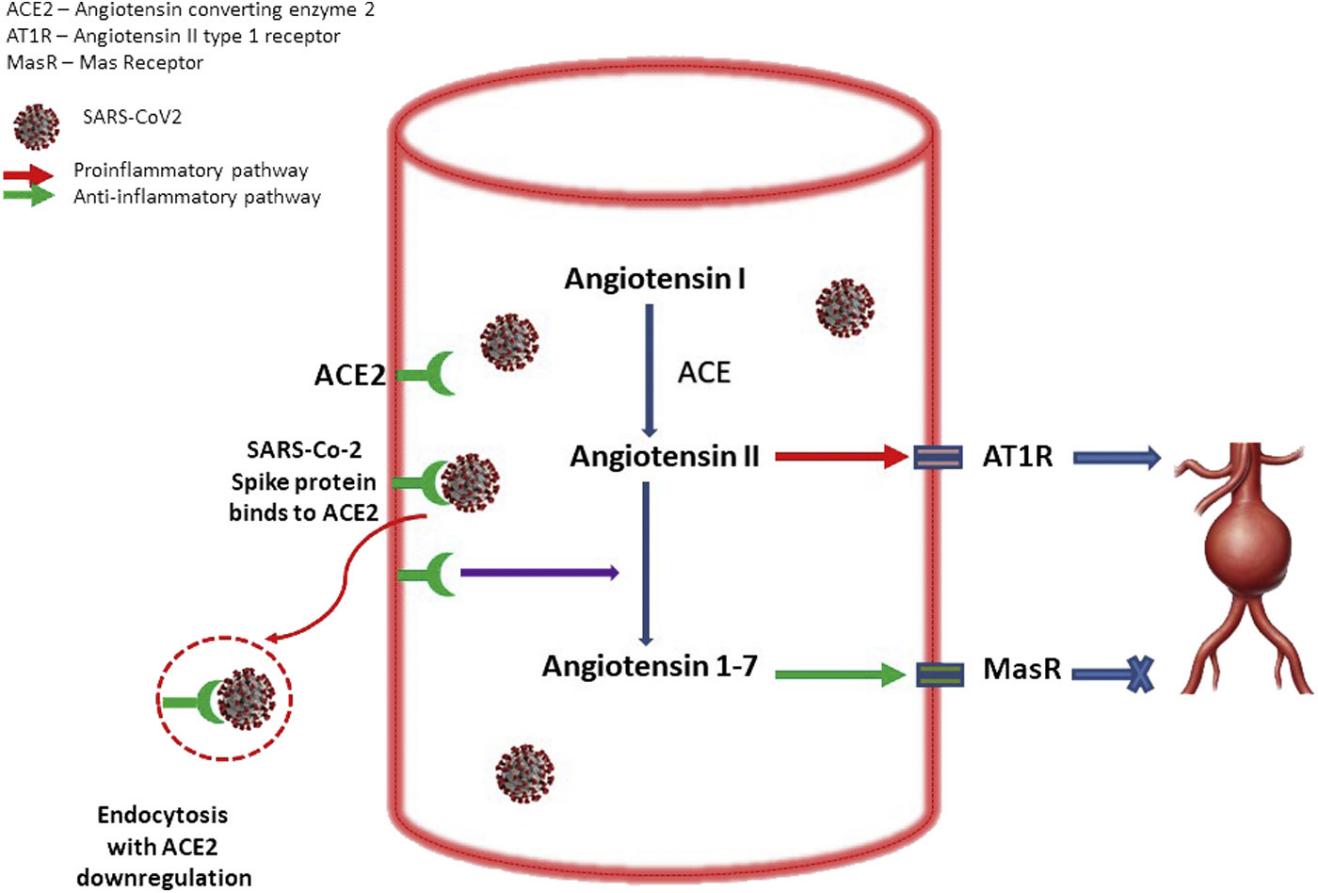
Pathogenesis of severe acute respiratory syndrome coronavirus 2 (SARS-CoV-2) and its effect on the angiotensin pathway by downregulating angiotensin-converting enzyme 2 (ACE2) is demonstrated. The subsequent effect on the angiotensin system resulting in increased activation of the proinflammatory angiotensin II type 1 receptor pathway to potentiate aneurysm growth is also illustrated.

The rapid increase in size of the aneurysm could, theoretically, have been linked to his COVID-19 infection. No other cause for pain or inability to flex the knee was noted in this patient. Furthermore, at 6 months after aneurysm exclusion, the patient's pain symptoms had resolved. These factors taken together suggest that the initial presentation was most likely related to the rapid expansion in the size of the popliteal artery aneurysm. Experimental evidence has proven that aneurysm pathogenesis is related to elevated angiotensinogen II levels and angiotensin II type 1 receptor elevation.^4^ Experimental aortic aneurysms can be induced in hyperlipidemic mice with elevated angiotensin II levels.^5^ ACE2 is an integral membrane protein that serves as the host cell entry point for the SARS-CoV-2 virus.^6^ This resulting downregulation of ACE2 (Fig 3) has been linked to increased angiotensin II levels in humans infected with the SARS-CoV-2 virus.^7^ Hence, it is possible that elevated angiotensin II levels in our patient infected with SARS-CoV-2 might have resulted in rapid aneurysm growth with the resulting acute pain and disability. Further studies are urgently needed to better understand whether upregulation of the angiotensin II/angiotensin II type 1 receptor proinflammatory axis by SARS-CoV-2 could influence aneurysm growth.

The second patient with spontaneous axillary artery thrombosis had had no previous thrombotic or embolic events and was found on further testing, as a part of a cardiology and coagulation specialist workup, to have normal echocardiography findings and a normal hypercoagulable panel. Additionally, the patient was not a smoker and had no other evidence of peripheral artery disease or extrinsic compression at the site of thrombosis. A noncovered stent was used to treat residual thrombus because it is my preference to use stents for nonaneurysmal lesions. Despite the computed tomography evidence of significant lung involvement and low oxygen saturation, the patient had no fever or elevated white blood cell count. Hence, SARS-CoV-2 infection would be the most likely etiology for the in situ thrombotic event. Various theories for the increased incidence of thrombotic events in patients with COVID-19 have been proposed, including (1) the formation of proinflammatory cytokines contributing directly to the rupture of the atherosclerotic plaque by local inflammation; (2) induction of procoagulant factors, and (3) systematic hypotension that predisposes to ischemia and thrombosis. Additionally, postmortem studies have demonstrated that SARS-CoV-2 virus has been found within the endothelial cells and had resulted in structural damage to the endothelium.^8^ Increased levels of D-dimer and CRP have also been observed in patients with COVID-19, and mortality and thrombotic events have been associated with higher levels.^9^ Both our patients had had elevated CRP and D-dimer levels, with a much more pronounced elevation in the patient with the axillary artery thrombosis (Table). It is as yet unknown whether these observed coagulation changes are a direct effect of SARS-CoV-2 or secondary to the cytokine storm.

No data are currently available specific to COVID-19 regarding the need for extended anticoagulation therapy. We decided on 6 months of oral anticoagulation therapy for the patient with the aneurysm owing to the decreased mobility on the affected leg and for the second patient because of COVID-19–related thrombosis. The use of newer anticoagulant drugs avoids the need for frequent international normalized ratio evaluations, which can pose additional risks and challenges of exposing others to infected patients and the lockdown of locations for international normalized ratio checks. The presented patients did not have any prohibitive risk factors for open surgical repair. The decision for endovascular procedures was primarily determined by the combined decision with the anesthesia team to avoid the use of general anesthesia. The use of percutaneous techniques minimizes the risk to healthcare workers compared with open procedures, which might require general anesthesia. The outcomes have also been shown to be better when intubation is not required in patients with COVID-19.^10^ In a single series (n = 20) involving open surgical revascularization for acute limb ischemia in patients with COVID-19, the in-hospital mortality was 40%.^11^

## Conclusion

The evolving COVID-19 pandemic and its potential vascular manifestations might be underestimated at present. Further studies on aneurysm growth in patients infected with SARS-CoV-2 and the role of long-term anticoagulation therapy for patients with catastrophic vascular emergencies are urgently needed.
